# Optimizing Propranolol Therapy for Infantile Hemangiomas: The Role of the Multidisciplinary Team

**DOI:** 10.7759/cureus.75806

**Published:** 2024-12-16

**Authors:** Mayu Yasuda-Koiwa, Tetsushi Ogawa, Atsushi Ogawa, Fumihiko Takizawa, Yuri Mukoyama, Shun Moriguchi, Akiko Kishi, Nobukazu Hayashi, Tsuyoshi Isojima

**Affiliations:** 1 Department of Pediatrics, Toranomon Hospital, Tokyo, JPN; 2 Department of Dermatology, Toranomon Hospital, Tokyo, JPN

**Keywords:** infantile hemangioma, multidisciplinary medical team, propranolol, safety, side effect

## Abstract

Background: Oral propranolol therapy is currently the first choice for infants with infantile hemangiomas (IHs) requiring systemic treatment. This study aims to evaluate the safety and effectiveness of oral propranolol therapy for IHs and to assess the role of a multidisciplinary medical team in supporting optimal treatment.

Materials and methods: Clinical data were retrospectively reviewed from medical records in 150 Japanese infants with IH treated with propranolol orally at Toranomon Hospital. Patients with problematic IH, such as tumor-type IH or IH with ulceration, were eligible for inclusion. Treatment was managed by a medical team consisting of pediatricians, dermatologists, pediatric nurses, pharmacists, and nutritionists. Patients' general conditions and vital signs, such as blood pressure, pulse rate, respiratory rate, and blood sugar, were closely monitored before, one hour, and two hours after drug administration.

Results: Close collaboration among multidisciplinary medical team members allowed for accurate patient evaluation, contributing to the early detection of side effects, even if asymptomatic. When side effects were suspected, pediatricians and dermatologists discussed the need to reduce or discontinue the medication. Of the 150 patients, no one experienced severe side effects. Although five cases (3.3%) were suspected of having mild side effects (i.e., hypotension, n = 3; hypotension and hypoglycemia, n = 1; inspiratory stridor, n = 1), treatment could be continued by adjusting the dosage. One hundred twenty patients have completed the oral propranolol therapy with successful outcomes.

Conclusions: This study provided additional evidence of the safety and effectiveness of oral propranolol therapy in 150 Japanese infants with IH. A well-functioning multidisciplinary medical team is essential for optimal patient treatment.

## Introduction

Infantile hemangiomas (IHs) are the most common vascular tumors in children [1,2]. They are diagnosed in 1.1%-2.6% of newborns [3] and in 4%-5% of infants [1,2,4,5]. Several racial differences have been reported in their incidence, with 8%-12% observed in Caucasian children [6] and 1.7% in Japanese children [7]. These findings indicate that genetic factors may also play a significant role in the development of IH [8].

Although several treatment options for IH are available, including corticosteroids, laser therapy, cryotherapy, and surgery, oral treatment with propranolol, a nonselective beta-blocker, has emerged as the most effective treatment for high-risk IH [2]. The effectiveness of propranolol therapy in infants with IH was first reported in 2008 [9]. In Japan, oral propranolol was approved for IH in 2016 [10,11] and is currently the first-line treatment for infants requiring systemic treatment [12].

Since propranolol is a beta-blocker, its use requires careful management of systemic effects, particularly side effects. Gomes et al. reported on the clinical investigation involving 118 patients with IH treated with oral propranolol and noted that a multidisciplinary team of experts is important to ensure appropriate management [13]. The role of multidisciplinary care in treatment should be emphasized more in clinical practices to provide safer and more effective treatments and to manage potential side effects. However, there are few reports focusing on this perspective.

In this study, we conducted a retrospective analysis of 150 infants who received oral propranolol therapy to evaluate the safety and effectiveness of the treatment and to assess the role of a multidisciplinary medical team in achieving optimal outcomes.

## Materials and methods

Study population

Clinical data were retrospectively reviewed from the medical records of 150 patients with IH who received oral propranolol therapy at Toranomon Hospital, Tokyo, Japan, between October 2016 and November 2023. Patients with problematic IH, such as tumor type IH or IH with ulceration, were eligible for inclusion. The definition of problematic IH aligns with the criteria for high-risk IH outlined in the Clinical Practice Guidelines by the American Academy of Pediatrics [2]. Moreover, despite being a plaque type, lesions with ulceration or functional impairment risk were classified as problematic [2]. Oral propranolol therapy was also recommended for patients with IH who might have cosmetic concerns after involuting. Patients with contraindications such as bradycardia, hypoglycemia, bronchial asthma, and atrioventricular block were not provided this treatment. All treated cases were included in the study, so no exclusion criteria were applied.

Before initiating the oral propranolol therapy, patients' vital signs were assessed, including blood pressure, pulse rate, respiratory rate, saturation of percutaneous oxygen, and blood glucose levels. They were also interviewed about their detailed perinatal, medical, and family history, natal environment, and feeding status (formula milk, breast milk, or mixed, volume per feeding, number of feedings per day, and number of solid foods per day). Additionally, all patients also underwent electrocardiography.

Oral propranolol therapy

Patients were admitted to the hospital to initiate oral propranolol therapy (Hemangiol syrup 0.375%, Maruho Co. Ltd, Osaka, Japan). Upon admission, a pediatrician conducted thorough examinations to ensure that patients were in good health. The initiation of oral propranolol therapy was comprehensively performed with the cooperation of board-certified pediatricians and dermatologists and dedicated pediatric nurses, pharmacists, and nutritionists working within the pediatric ward.

Propranolol was prescribed based on the infant’s weight. The recommended starting dose was 1 mg/kg/day, divided into two doses for the first two days, then increased to 2 mg/kg/day for the following two days, and subsequently to 3 mg/kg/day [2,9,10,14]. The therapeutic dose was 3 mg/kg/day, administered in two doses (once in the morning and once in the evening), with at least a nine-hour interval. The medication was administered immediately after feeding or eating. Vital signs, such as blood pressure, pulse rate, respiratory rate, and blood glucose levels, were monitored before, one hour, and two hours after each administration. Blood glucose levels were measured using Medisafe Fit (Terumo Corporation, Tokyo, Japan). After the two-hour observation period, patients were closely monitored, with vital signs measured at three- to eight-hour intervals. Continuous electrocardiogram monitoring was maintained throughout their hospital stay. The criteria for evaluating vital signs were based on the US and UK attachments of Pierre Fabre Dermatologie regarding hypotension and bradycardia. Hypoglycemia was defined as a blood glucose level of 3.9 mmol/L (70 mg/dL), with levels below 3.0 mmol/L (54 mg/dL) considered clinically severe hypoglycemia, as they may cause neurogenic symptoms and cognitive dysfunction [15].

Once the appropriate maintenance dose was established during hospitalization, the patient was followed up in the outpatient department by our team. At each visit, the progression of IH was assessed clinically and photographically. Oral propranolol therapy usually ended after six months but was extended in cases where the dermatologist determined additional treatment was necessary, such as when tumors persisted or remained in the growth phase.

Assessment of clinical response

Pre- and posttreatment examinations and evaluations of IH were performed by a single skilled dermatologist. Each IH was evaluated for surface appearance, including color, volume, and the presence of ulcer. The location of the IH was also examined using pre- and posttreatment photographs. Tumor volume was calculated based on length, width, and depth measurements. Changes in the color tone of IH during the early stages were analyzed using photographs taken at each volume evaluation. Treatment success was defined as complete or nearly complete resolution of the target IH by week 24, according to a previous report [10]. Nearly complete resolution was characterized by minimal telangiectasia, erythema, skin sickening, soft tissue swelling, and/or distortion of anatomical landmarks [10].

## Results

Induction of oral propranolol therapy through a multidisciplinary medical team

**Figure 1:**
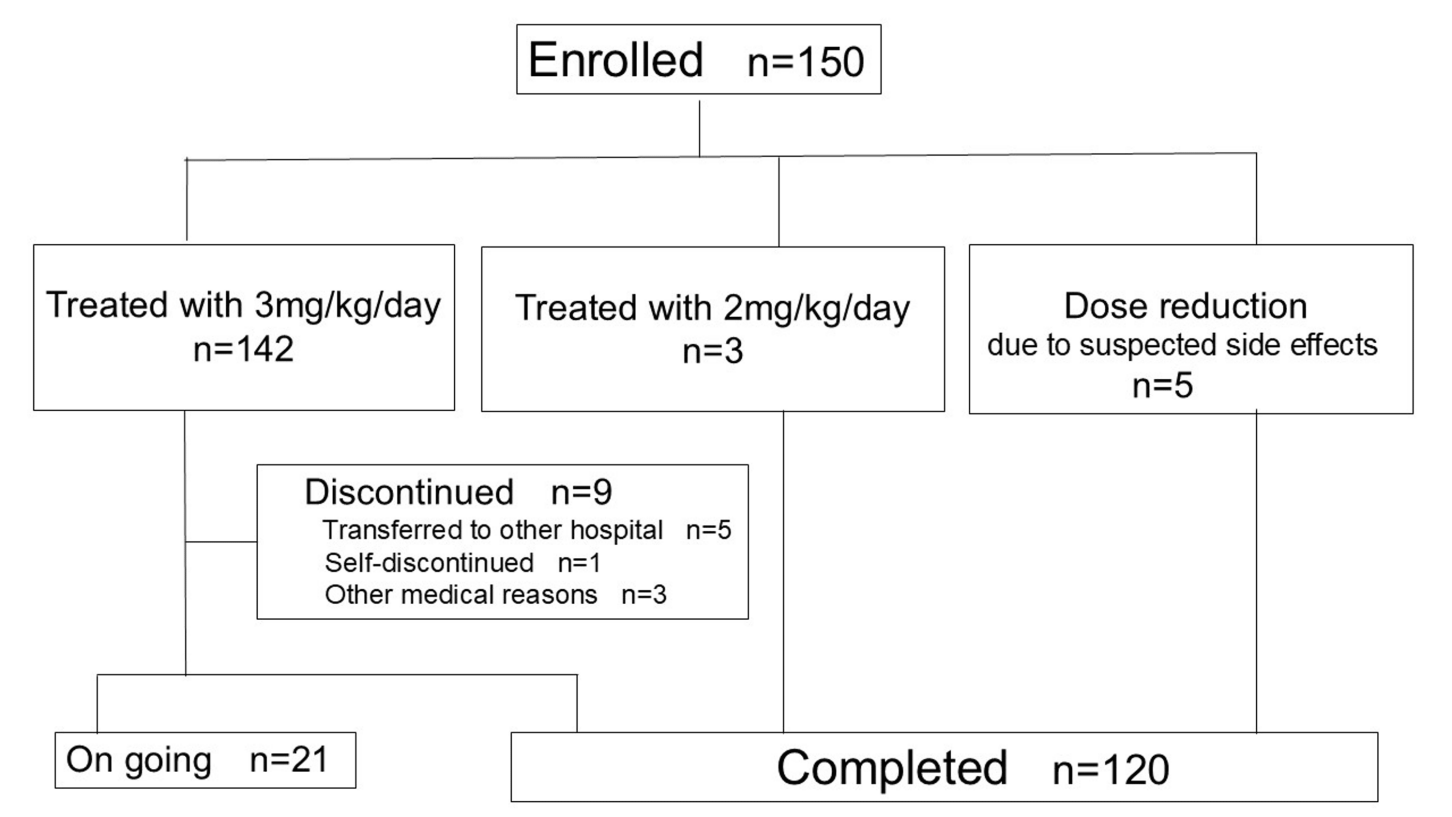
Flow diagram of oral propranolol therapy for IH cases

**Figure 2:**
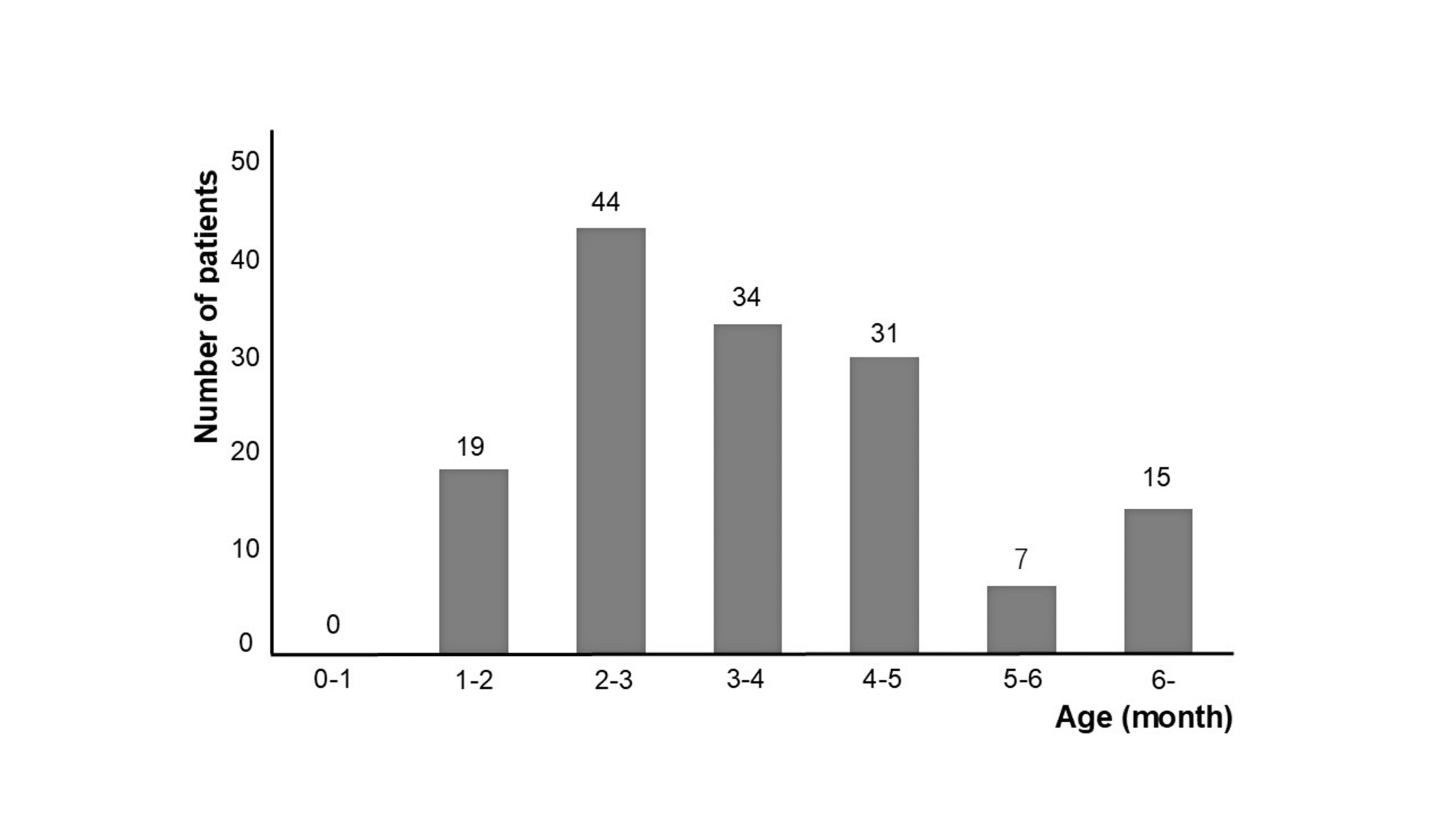
Starting age for treatment

Figure 1 shows the flow diagram of oral propranolol therapy for our IH cases, and Figure 2 presents the starting age for treatment.

**Figure 3:**
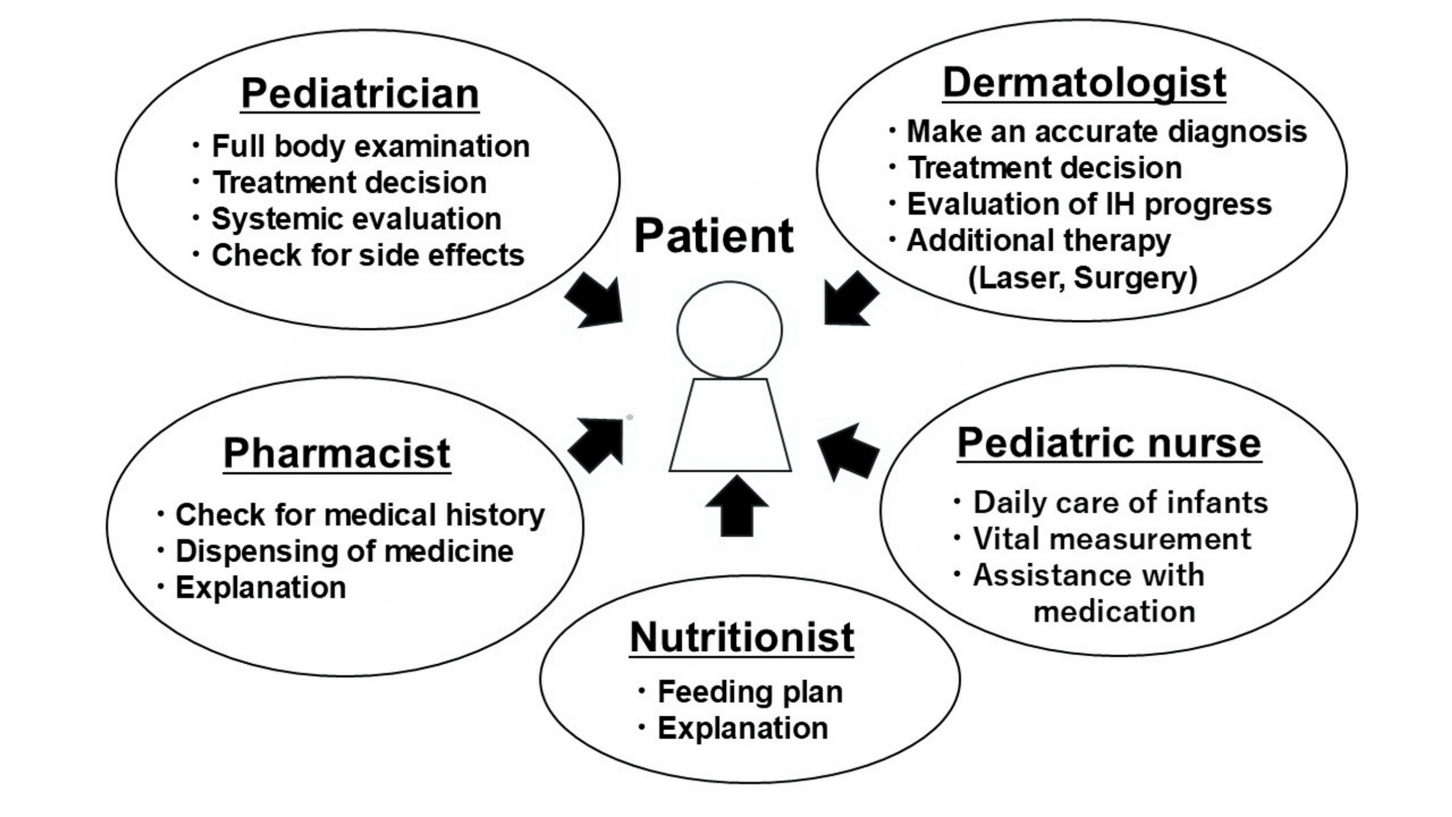
The multidisciplinary medical team at our hospital Image credit: Authors.

For these patients, a multidisciplinary team collaborated to facilitate the induction of oral propranolol therapy, as shown in Figure 3. 

The dermatologist initially diagnosed the skin lesion as an IH with accuracy. All the cases were definitively diagnosed by visual inspection, and no case required ultrasound or MRI, even those involving subcutaneous lesions. When oral propranolol therapy was deemed necessary, the dermatologist consulted closely with the pediatrician, who then performed a systemic evaluation to determine whether propranolol treatment was appropriate. Thus, the dermatologist and pediatrician jointly made the final treatment decision for each individual. Pediatric nurses, experienced in infant care and vital sign monitoring, administered the medications. Close collaboration between the nurses, the pediatricians, and the dermatologists ensured accurate patient evaluations, which enabled the early detection of side effects, even if asymptomatic. In cases where side effects were suspected, the dermatologists and the pediatricians discussed the need to adjust or discontinue the medication. The pharmacist assigned to the pediatric ward reviewed each patient's medical history and provided families with clear instructions and precautions, supported by an easy-to-understand pamphlet. Dispensing propranolol was a meticulous process: it was double-checked by two pharmacists, and another two pharmacists inspected the prepared medication. Among the 60 pharmacists working at our hospital, only 10, who were specifically trained in propranolol preparation, were authorized to dispense the drug. This system ensured the accurate preparation of small doses. The nutritionist created tailored feeding plans, particularly to prevent hypoglycemia, consulting closely with families. For low-birthweight or premature infants, frequent feedings were recommended, while for children eating solid foods, strategies were provided to manage longer nighttime intervals between meals. Detailed advice on feeding schedules and frequency was individualized for each patient.

All professionals convene weekly for a conference to discuss IH cases. Pediatricians and pediatric nurses are always onsite in the pediatric ward, overseeing treatment and evaluation. Dermatologists, pharmacists, and nutritionists checked the patient's condition using electronic medical records and visited the ward daily to provide care through face-to-face consultations with pediatricians and pediatric nurses.

Side effects

Of the 150 patients, 142 received a maintenance dose of 3 mg/kg/day of propranolol during hospitalization. In five patients, the dose was reduced to 1.0 or 1.5 mg/kg/day due to suspected side effects, including three patients with hypotension, one with hypotension and hypoglycemia, and one experiencing stridor immediately after administration. Details of the suspected side effects are summarized in Table 1.

**Table 1:** Suspected side effects observed in five patients

Patient number	Sex	Past medical history/comorbidity	Starting age	Side effects	Drug dose when side effects occur (mg/kg/day)	Final adjusted dose (mg/kg/day)	Response
1	Male	None	Two months	Hypotension (65/31 mmHg)	2	1	Effective
2	Female	PHACE syndrome suspected premature, low birthweight	Three months, 15 days	Hypotension (69/34 mmHg)	2	1.5	Effective
3	Female	None	Five months, six days	Hypotension (68/42mmHg)	2	1	Effective
4	Female	PELVIS syndrome	One month, 30 days	Hypotension (63/28 mmHg), hypoglycemia (54 mg/dL)	2	1.5	Effective
5	Female	Neonatal respiratory failure	One month, 16 days	Stridor	2	1	Effective

All patients with hypotension and hypoglycemia were asymptomatic, which was observed on the day the dose was increased to 2 mg/kg/day but resolved spontaneously with follow-up, without requiring intervention. The five affected patients continued treatment at reduced doses (1 mg/kg/day for three patients and 1.5 mg/kg/day for two patients) and achieved good therapeutic outcomes. Among these cases, one patient was hospitalized for nine days due to dosage adjustments, while the remaining four were discharged within the standard six-day period. In another three patients, relatively low blood pressure or blood glucose levels were noted before initiating propranolol therapy at the scheduled dose of 3 mg/kg/day. Consequently, the dose was set at 2 mg/kg/day, which had been well-tolerated without issues on the previous day.

Demographics

Table 2 shows the demographic data of the infants.

**Table 2:** Demographic data, IH type, and IH location IH: infantile hemangioma.

	Background	N (%)
Sex	Male	29 (19.3)
	Female	121 (80.7)
Gestational age at birth	Preterm (<37 weeks)	20 (13.3)
	Term (37 ≦ 42 weeks)	129 (86.0)
	Postterm (≧42 weeks)	0 (0.0)
	Unknown	1 (0.7)
Birthweight	<2500 g	25 (16.7)
	2500 ≦ 4000 g	121 (80.7)
	Unknown	4 (2.6)
IH type	Tumor	74 (49.3)
	Subcutaneous	4 (2.7)
	Plaque	15 (10.0)
	Tumor and subcutaneous	18 (12.0)
	Plaque and subcutaneous	39 (26.0)
IH location	Head and neck	98 (65.3)
	Shoulder and chest	15 (10.0)
	Abdomen	8 (5.3)
	Genital area	3 (2.0)
	Hips and back	7 (4.7)
	Buttocks	1 (0.7)
	Extremities	18 (12.0)
	Total cases	150 (100.0)

A total of 150 infants (29 boys and 121 girls) were enrolled, with approximately four times more girls than boys. Among these, 20 infants were born preterm (before 37 weeks), and 25 had low birthweight (≤2500 g).

The types and locations of IH are also described in Table 2.

Three primary types (tumor, subcutaneous, and plaque) and two mixed types (tumor-subcutaneous and plaque-subcutaneous) were noted. The tumor type (49.3%) was predominant, followed by the subcutaneous-plaque mixed type (26.0%) and the tumor-subcutaneous mixed type (12.0%). The plaque type alone accounted for 10.0%.

Regarding the location of IH, also shown in Table 2, the head and neck regions were the most frequent sites, comprising 65.3% of all IH cases, followed by the shoulder and chest (10.0%) and the abdomen (5.3%).

Of the 150 cases, 147 met the American Academy of Pediatrics Clinical Practice Guideline criteria for high-risk IH requiring treatment [2]. These criteria include facial IH ≥ 2 cm (≥1 cm if ≤3 months of age); scalp IH > 2 cm; neck, trunk, or extremity IH ≥ 2 cm; and thick superficial IH ≥ 2-mm thickness.

Clinical outcomes

During the outpatient follow-up period, nine cases discontinued treatment at our hospital, as shown in Figure 1. Of these, five patients were transferred to another hospital due to relocation, one self-discontinued, and three stopped treatments for other medical reasons. Two of these three patients had repeated colds, and one was diagnosed with bronchial asthma. The patient with bronchial asthma experienced residual scarring and underwent excision surgery at age five.

To date, 120 patients have completed the oral propranolol therapy at our hospital, with successful outcomes in all cases. Treatment was discontinued once complete or nearly complete resolution was achieved, as previously described. Although the observation period after the end of treatment varied for each case, patients were monitored monthly for at least three months to check for re-growth, with subsequent follow-up intervals determined on a case-by-case basis. Of the 120 patients, 17 required additional laser therapy for residual erythema, and three were re-administered oral propranolol treatment due to tumor re-growth.

## Discussion

This study highlights the crucial role of our well-coordinated multidisciplinary team approach in providing safe and effective medical care for patients with IH. Figure 3 shows the medical team comprising various professionals at our hospital. These collaborations are particularly effective in preventing and detecting side effects at an early, often asymptomatic stage.

While most side effects of propranolol are minor, such as diarrhea [10], serious side effects such as hypotension, bradycardia, and hypoglycemia have been reported and must be prevented [14]. Frequent monitoring of vital signs by pediatric nurses during hospitalization allowed for the early detection of asymptomatic hypotension and hypoglycemia. This vigilance also facilitated the determination of an optimal drug dose for each patient. Treatment was successfully completed in some cases by reducing the maintenance dose to 1.0 or 1.5 mg/kg/day after detailed consultations between dermatologists and pediatricians, which was supported by several reports indicating that even low doses of propranolol can effectively alter the color tone and growth of IH [16-18].

In the outpatient setting, routine assessment of vital signs is challenging; therefore, pediatricians must thoroughly educate families on the warning signs of bradycardia, hypotension, and hypoglycemia, such as weakness, cold hands and feet, pale complexion, and loss of consciousness. Pediatricians must instruct caregivers to discontinue taking propranolol immediately if the patient is unwell, has an empty stomach, or exhibits inadequate food intake, as poor feeding and infections are significant risk factors for hypoglycemia [14,19,20]. Severe hypoglycemia is more common at ≥1 year old due to extended overnight fasting following weaning [2,9,14,20]. Additionally, fasting for more than eight hours increases the risk of hypoglycemia in infants [14]. In this study, although 44 (36.7%) infants continued oral propranolol therapy beyond one year of age, none of them exhibited symptomatic hypoglycemia, as pediatricians consistently emphasized the importance of adequate feeding during every visit. Even during brief outpatient visits, pediatric nurses assessed vital signs. Since the amount of propranolol prescribed is determined by the infant's weight, pharmacists carefully monitored the dispensing, which changed with each visit. During the six-month follow-up period, infant feeding and dietary habits changed significantly, so nutritionists provided nutritional guidance tailored to the child's situation.

The optimal dose of propranolol is reported to range from 1 to 3 mg/kg/day [2,14], with many recommending an average dose of 2 mg/kg/day [14]. In Japan, the standard maintenance dosage has been set at 3 mg/kg/day [10], as specified in the propranolol medical package insert by the Pharmaceuticals and Medical Devices Agency, implying that more attention should be paid to side effects. Therefore, we initiated treatment in an inpatient setting to ensure careful monitoring, despite evidence that oral propranolol can be safely administered in outpatient settings [13,14].

Most of our demographic data were consistent with previous studies. Among the 150 patients enrolled, 121 were girls and 29 were boys, consistent with prior reports showing a higher prevalence in girls [5]. The most common IH site was the head and neck (65.3%), similar to previous reports [21]. In this study, tumor, subcutaneous, and mixed types (tumor-subcutaneous and plaque-subcutaneous) accounted for 135 patients (90.0%) of the total, while 15 cases (10%) of plaque-type were included. Despite being a plaque type, lesions with ulceration or functional impairment were classified as high risk and required early treatment induction [2]. In our cases, eight patients had ulceration, and four had lip lesions that were considered to have the potential to cause eating disorders, whereas the others were prominent or huge lesions on the face.

Although the treatment strategy and diagnostic criteria were consistent, the limitations of the present study include the retrospective design, lack of long-term follow-up, and a single-center setting. Further longitudinal studies will be needed to confirm the potential late relapses or delayed side effects. Because of a single-center study, there may be bias in patient selection. However, as many patients are referred to our institution from a wide area, we have a large number of patients with various types of IH. Another limitation would be the lack of comparative studies with other treatments, such as laser therapy or cryotherapy. In addition, this study did not examine combination therapy with pulsed dye laser therapy. Recently, several reports have suggested combining propranolol therapy with pulsed dye laser therapy resulting in earlier regression of IH and reducing cumulative propranolol doses, potentially decreasing serious treatment side effects and increasing efficacy [22-24]. However, the effects and side effects of combination therapy have not yet been fully explored; thus, further prospective investigations are needed. 

The strengths of this study lie in its analysis of the largest number of Japanese children with IH in a single institute, confirmation of all side effects, treatment completion even in those suspected of side effects by dose reduction, and review of the pediatrician’s perspective.

## Conclusions

This study provided additional supporting the safety and effectiveness of oral propranolol therapy in 150 Japanese infants with IH. A well-coordinated multidisciplinary team plays an essential role in preventing severe side effects, effectively managing mild side effects, and achieving successful treatment outcomes.
